# Rheological and Gelling Properties of Chicken-Mushroom Hybrid Gel for Flexitarian-Friendly Functional Food Applications

**DOI:** 10.3390/foods14040645

**Published:** 2025-02-14

**Authors:** Ngassa Julius Mussa, Manat Chaijan, Porntip Thongkam, Chantira Wongnen, Warangkana Kitpipit, Hasene Keskin Çavdar, Siriporn Riebroy Kim, Worawan Panpipat

**Affiliations:** 1 Food Technology and Innovation Research Center of Excellence, School of Agricultural Technology and Food Industry, Walailak University, Nakhon Si Thammarat 80160, Thailand; ngassajulius@gmail.com (N.J.M.); cmanat@wu.ac.th (M.C.); pohntip23@gmail.com (P.T.); chantira.wo@wu.ac.th (C.W.); warangkana.ki@wu.ac.th (W.K.); 2 Akkhraratchakumari Veterinary College, Walailak University, Nakhon Si Thammarat 80160, Thailand; 3 Department of Food Engineering, Faculty of Engineering, Gaziantep University, TR-27310 Gaziantep, Turkey; hasenekeskin@gantep.edu.tr; 4 Food and Nutrition Program, Faculty of Agriculture, Kasetsart University, Bangkok 10900, Thailand; siriporn.r@ku.th

**Keywords:** split gill mushroom, hybrid meat, chicken, gel, sustainability

## Abstract

Hybrid gels combining chicken and mushroom offer innovative functional food choices, catering to the growing demand for flexitarian-friendly products. These gels reduce meat content while enhancing dietary fiber, bioactive compounds, and sustainability. This study examined the effects of split gill mushroom (*Schizophyllum commune*) powder (SGM) substitution (0%, 25%, 50%, and 75%, *w*/*w*) for Ligor chicken meat in hybrid gels, focusing on rheological and gelling properties. The 25% SGM gel demonstrated optimal performance in terms of rheology, texture, microstructure, pH, water-holding capacity, and color. At this level, hybrid gels exhibited superior gelation properties, demonstrating elasticity dominance, as indicated by a higher storage modulus (G′) than loss modulus (G″), along with stable cohesiveness and unaffected springiness (*p* > 0.05). However, hardness, gumminess, and chewiness were significantly lower than the control (*p* < 0.05). Higher SGM levels (50–75%) markedly weakened the gels, reducing viscoelasticity, increasing porosity and water release, and causing discoloration. These findings highlight 25% SGM as an optimal level for hybrid meat gels, maintaining product quality while promoting sustainability in the meat industry.

## 1. Introduction

Meat is a major source of high-quality protein, vitamins (thiamin, riboflavin, niacin, pantothenic acid, B6, and B12), and minerals like iron, zinc, and phosphorous [1]. However, the production and consumption of meat have raised concerns due to their negative effects on the environment, public health, and animal welfare [2,3]. From an environmental perspective, the emission of greenhouse gases from animal farming activities has contributed to climate change. About 50–75% of greenhouse gases in particular methanic emission are from livestock [4,5]. Additionally, excessive consumption of red meat and processed meat is associated with an increased risk of chronic illnesses, such as cardiovascular diseases and diabetes [6].

To address these challenges, researchers have proposed innovative solutions like hybrid meat products, representing a fascinating intersection of food science and consumer trends. By combining animal-based and plant-based ingredients, these products aim to reduce the environmental footprint of meat while maintaining consumer satisfaction [7]. Hybrid meat products are formulated by replacing a certain proportion of meat, typically ranging from 20 to 50% or more, with plant components rich in proteins such as fruits and vegetables, to create more nutritionally balanced foods [8,9]. These products offer a convenient way to reduce meat consumption without drastically altering dietary preferences or scarifying taste [10]. Furthermore, hybrid meat aligns with sustainability goals by conserving biodiversity, land, water, and energy while supporting animal welfare [10,11]. This strategy is ideal for the expanding flexitarian trend, in which people cut back on meat consumption without giving it up entirely [12].

Healthy meals with high protein content and no meat, such as soy, wheat, mycoprotein, or mushroom protein, have sparked global attention. Mushrooms have gained popularity among non-meat-based ingredients due to their outstanding nutritional profile. Edible mushrooms are high in protein, which includes all essential amino acids, as well as dietary fiber, non-starchy carbohydrates, vitamins, and minerals [13,14]. They additionally consist of bioactive compounds with antioxidative, anti-inflammatory, and immunomodulatory activities [15,16]. Incorporating mushrooms into meat-based products has been proven to improve sensory quality, texture, emulsion stability, and shelf life while decreasing lipid content and cooking loss [7,17,18]. 

*Schizophyllum commune*, also known as the split gill mushroom (SGM), is an edible Basidiomycete. This rubber wood-decaying fungus can be found in multiple locations throughout the world and is widely distributed throughout Asia’s rainforests, especially in Southeast Asian countries like Southern Thailand, due to its taste and therapeutic properties [19,20,21,22]. SGM is famous in Asia because of its umami taste, which resembles meat [23]. SGM has long been utilized as a favored protein substitute for animal proteins due to its higher content of key amino acids than vegetables [24]. It has been found that compared to other vegetables, SGM has more important amino acids, such as lysine, leucine, arginine, threonine, and valine, which account for around 41% of the total amino acid composition [24].

SGM’s nutritional value can be ascribed to its high protein (about 24.5%) and fiber (around 20%) content, as well as minerals such as iron (approximately 2730 mg/kg), zinc (about 188 mg/kg), and manganese (around 64 mg/kg), but its fat content is relatively low (roughly 1.3%) [25]. SGM has historically been used as medicine due to its fruiting body’s biologically active components, including phenolic compounds, vitamins, carboxylic esters, and β-glucan polysaccharide, notably schizophyllan [19,24,25]. These bioactive compounds may have potential health benefits, such as immune system activation [26], antioxidant action [27], anticancer activity, inflammation suppression [28], antidiabetic activity, and antimicrobial activity [19,25]. As a result, this mushroom and its isolated fractions have become widely used in the cosmetic, medical, pharmaceutical, nutraceutical, and food industries.

Ligor chicken is a promising new breed developed in Thailand that combines the flavor and nutritional value of native Dang Suratthani chickens with the rapid growth rate of SUT 101 crossbred parents [29]. The new combination results in a hybrid chicken with improved growth performance, leaner flesh with higher protein content, and a unique amino acid profile [29]. While heat-induced gelation of Ligor chicken meat has been documented [30], the innovative product from this chicken meat can be extended by hybridizing with plant-based ingredients, paving the way for combining Ligor chicken meat with plant-based ingredients to create a healthier hybrid meat product. Despite the reported impacts of other mushrooms on hybrid meat characteristics, there is limited information on how SGM affects Ligor chicken meat gel functionality. As a result, the purpose of this study was to determine the influence of different levels of SGM powder on the rheological and gelling properties of Ligor chicken-mushroom hybrid gels. By combining Ligor chicken meat and SGM, the resulting hybrid gels can be used as an additional food option, increasing the possibility of using both Ligor chicken and SGM as culinary components.

## 2. Materials and Methods

### 2.1. Chemical 

All chemicals and reagents utilized in this study such as glutaraldehyde, ethanol, and tert-butyl alcohol were purchased from Sigma-Aldrich (St. Louis, MO, USA).

### 2.2. Preparation of Split Gill Mushroom (Schizophyllum commune) (SGM) Powder

**Figure 1:**
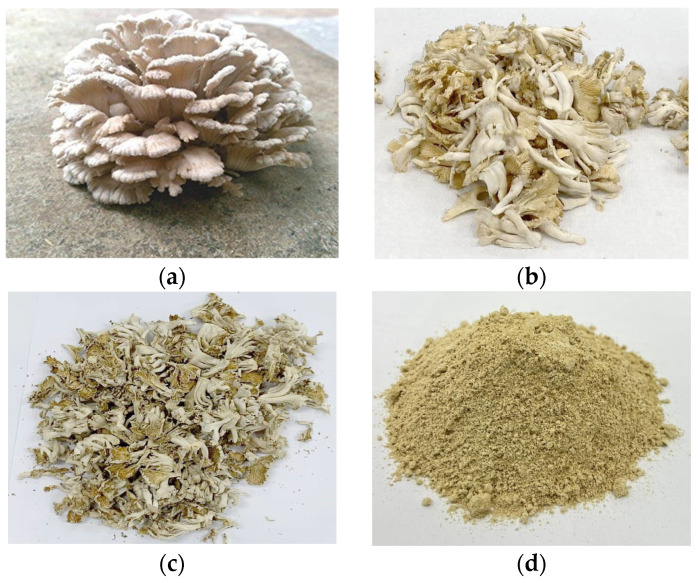
Whole fresh split gill mushroom (*Schizophyllum commune*) (SGM) (**a**), freshly prepared SGM (**b**), dried SGM (**c**), and SGM powder (**d**).

Freshly harvested edible SGM (5 kg) were bought from local fresh market in Tha Sala District, Nakhon Si Thammarat, Thailand (Figure 1a,b). To prepare the SGM powder, methods from previously published studies were adopted [31,32,33]. At first, raw SGM was washed with potable water several times to remove dirt, residual compost, and any foreign materials. The cleaned mushrooms were spread in a single layer on stainless steel trays to ensure uniform drying. Drying was performed using a hot air oven (Memmert UF260, Schwabach, Germany) set at 60 °C for 48 h, until the SGM reached a final moisture content of approximately 7% (*w*/*w*) (Figure 1c). The drying temperature was controlled to minimize the decomposition and/or oxidation of bioactive compounds, ensuring the retention of their key functional properties. Then, the dried SGM was ground into a fine powder using a grinder (Panasonic MK—G20MR, Japan) and passed through a 20-mesh sieve to ensure uniformity (particle size~850 µm) (Figure 1d). The SGM powder was immediately vacuum-packed in airtight polyethylene bags to prevent moisture absorption and oxidation. The packaged powder was stored at room temperature (25–27 °C) and used within 1 month to maintain its quality.

### 2.3. Collection and Preparation of Chicken Meat 

The 12-week-old Ligor chicken carcasses, which weighed 1.5 ± 0.1 kg and were acquired from Smart Farm Walailak University in Nakhon Si Thammarat, Thailand, were packed in an ice-filled polystyrene box and brought to the lab in within 30 min. Upon arrival, the breast and thigh without skin were separated from the carcasses and mixed at a 1:1 (*w*/*w*) ratio to create composite meat. The composite meat was chopped by a Talsa Bowl Cutter K15e (The Food Machinery Co., Ltd., Kent, UK) to create a homogeneous composite sample. This study employed three separate composite samples from independent batches.

### 2.4. Hybrid Meat Gel Preparation

**Figure 2:**
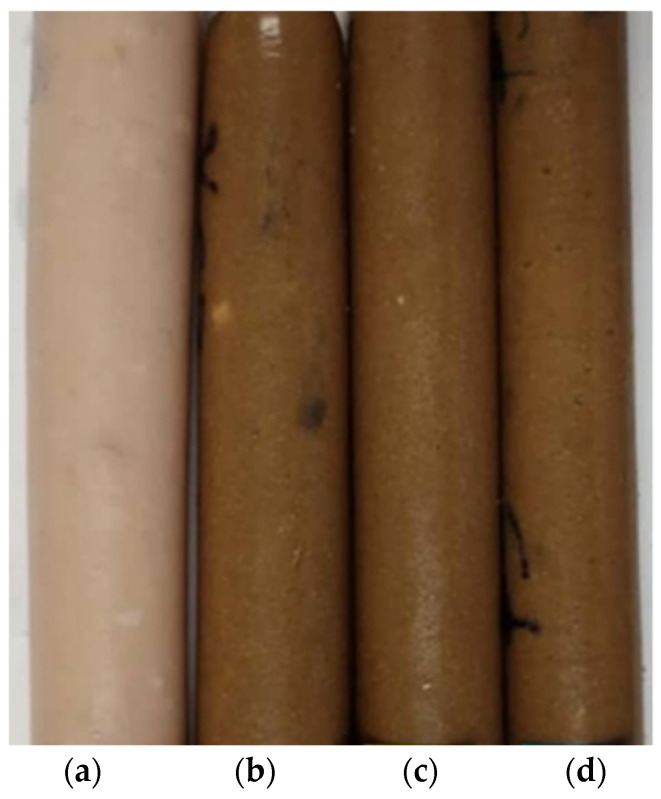
Visual representation of chicken-mushroom hybrid sols with varying levels of spilt gill mushroom (*Schizophyllum commune*) (SGM) powder substitution before thermal gelation: (**a**) Control sample (0% SGM); (**b**) 25% SGM substitution; (**c**) 50% SGM substitution; and (**d**) 75% SGM substitution.

In this study, chicken-mushroom hybrid gel formulations were created with varying SGM powder substitution percentages. Each of them had either 0 (control), 25, 50, or 75% (*w*/*w*) SGM powder, which meant that the Ligor chicken meat was replaced with 0, 25, 50, or 75% SGM powder based on the overall weight of the meat sample. In the gel model system without seasoning, except for salt to solubilize myofibrillar proteins, minced Ligor chicken meat sample was chopped with 2.5% (*w*/*w*) salt for 3 min using a Talsa Bowl Cutter K15e. Then, SGM powder was added and chopped for 3 min to produce a homogeneous sol. Afterward, the sols were divided into two groups. The first groups were rheologically analyzed, while the second groups were stuffed into casings (2 cm in diameter; Figure 2) and cooked in two steps (40 °C/30 min followed by 90 °C/20 min) to generate thermally induced gels. To maintain food safety, the product’s core temperature must be at least 72 °C. After heating, the gels were cooled in cold water for 30 min and stored at 4 °C for 24 h (overnight) [30]. The gelation functionality of the hybrid gels was then assessed, including texture profile analysis (TPA), microstructure, pH, expressible fluid, and color. 

### 2.5. Rheological Analysis

Rheological properties of the hybrid sol were determined according to the method of Somjid et al. [34], using HAAKE MARS 60 Rheometer (Thermo Fisher Scientific Inc., Yokohama, Japan), in order to monitor the tendency of gel-forming ability. Briefly, approximately 0.5 g sol was spread on the sample holder under a 35 mm parallel plate geometry. The gap between the plate and the sample holder was fixed at 0.5 mm, and a thin layer of silicone oil was added to prevent dehydration. Heating was performed at a constant frequency of 1 Hz and an amplitude strain of 2%, which was tithing the linear viscoelastic region. The rheological test conditions were selected based on those reported in the literature for similar gel products [34]. The determination of the linear viscoelastic region (LVR) was performed by conducting an amplitude sweep test prior to the frequency sweep [34]. Based on the literature and the results of the LVR test, a strain amplitude of 2% was chosen, as it was within the range where the sample exhibited stable rheological behavior. The temperature was swept from 22 °C to 90 °C at a rate of 3 °C per min, and the variations in rheological parameters, such as elastic or storage modulus (G′), viscous or loss modulus (G″), and tan δ, were recorded. To ensure accuracy and dependability, the rheometer was calibrated prior to each measurement following a regular protocol. The instrument was zeroed, and the measurement system was verified with a standard reference material, in this case silicone oil, to ensure that the expected viscosity or modulus values were obtained. Additionally, a temperature calibration was performed to maintain precise control during the analysis. The control sample (without SGM powder) served as an internal reference for comparison across treatments.

### 2.6. Gel Analysis

#### 2.6.1. Textural Profile Analysis (TPA)

The gels’ TPA were carried out as described by Li et al. [35], utilizing a texture analyzer (TA-XT2i, Stable Micro Systems Ltd., Godalming, UK) equipped with a cylindrical probe (P/50, 50-mm stainless cylinder) and a 25-kg load cell. The samples were cut into cylindrical forms (2.0 cm depth and 2.5 cm diameter) for TPA. A double compression cycle test was performed up to 50% compression of the original height. The time between the two compression cycles was 1 s. The trigger force employed in the test was 5 g, with a test speed of 5 mm/s. Following the test, the TA-XT Express software (version 19.0) computed and reported the TPA parameters for hardness (the maximum force required to compress the sample), springiness (the ability of the sample to recover its original form after deforming force has been removed), cohesiveness (the amount of deformation the sample could undergo prior to rupture), gumminess (the energy required to ingest semisolid food), and chewiness (the amount of work required to chew a solid sample to a steady state of swallowing).

#### 2.6.2. Microstructure Analysis

A scanning electron microscope (SEM) (Gemini SEM, Carl Ziess Microscopy, Oberkochen, Germany) was used to examine the microstructures of gels at a 10 KV acceleration voltage [13,36]. Briefly, the gel samples were cut into cubes (3 × 3 × 3 mm^3^) and washed using a phosphate buffer solution (0.2 M, pH 7.0) to eliminate dust and impurities from the sample surface. The washed samples were then fixed using a 2.5% glutaraldehyde solution for 10 h. The fixed samples were washed three times with the same buffer, dehydrated using 30, 50, 70, and 90% ethanol solutions, and then dehydrated three times in 100% ethanol; each dehydration step was performed for 10 min. The dried gel samples were treated three times with tert-butyl alcohol for 15 min each. Finally, the gel samples were mounted on a sample table and coated with 10 nm gold film using an ion sputtering apparatus (SC100, Hitachi, Tokyo, Japan).

#### 2.6.3. pH Determination

The pH of the gels was determined after homogenizing the gel sample with distilled water at a 1:10 weight-to-volume ratio, with a pH meter (EUTECH PH700, Singapore).

#### 2.6.4. Expressible Fluid Determination

A 0.5 cm thick gel sample was weighed and placed between two pieces of Whatman filter paper No. 1 at the top and three pieces of the same filter paper at the bottom. The standard weight (5 kg) was placed on top of the sample and kept there for 2 min. The sample was then taken out and weighed again. The expressible fluid was determined and represented as a percentage of sample weight [34].

#### 2.6.5. Color Analysis

Colorimetric values of gels, including L* (lightness), a* (redness/greenness), and b* (yellowness/blueness), were examined with a portable Hunterlab Miniscan/EX instrument (10° standard observers, illuminant D65; Hunter Assoc. Laboratory, Reston, VA, USA) [34].

### 2.7. Statistical Analysis

For each parameter, data were collected in three replications. Statistical analyses were performed using the SPSS software (version 16.0; SPSS Inc., Chicago, IL, USA). The one-way ANOVA was used to compare the means of chicken gel substituted with SGM powder at different concentrations. The results are presented as the mean ± standard deviation of the mean (SD) at a *p*-value < 0.05, which differed significantly.

## 3. Results and Discussion

### 3.1. Dynamic Rheological Properties 

**Figure 3:**
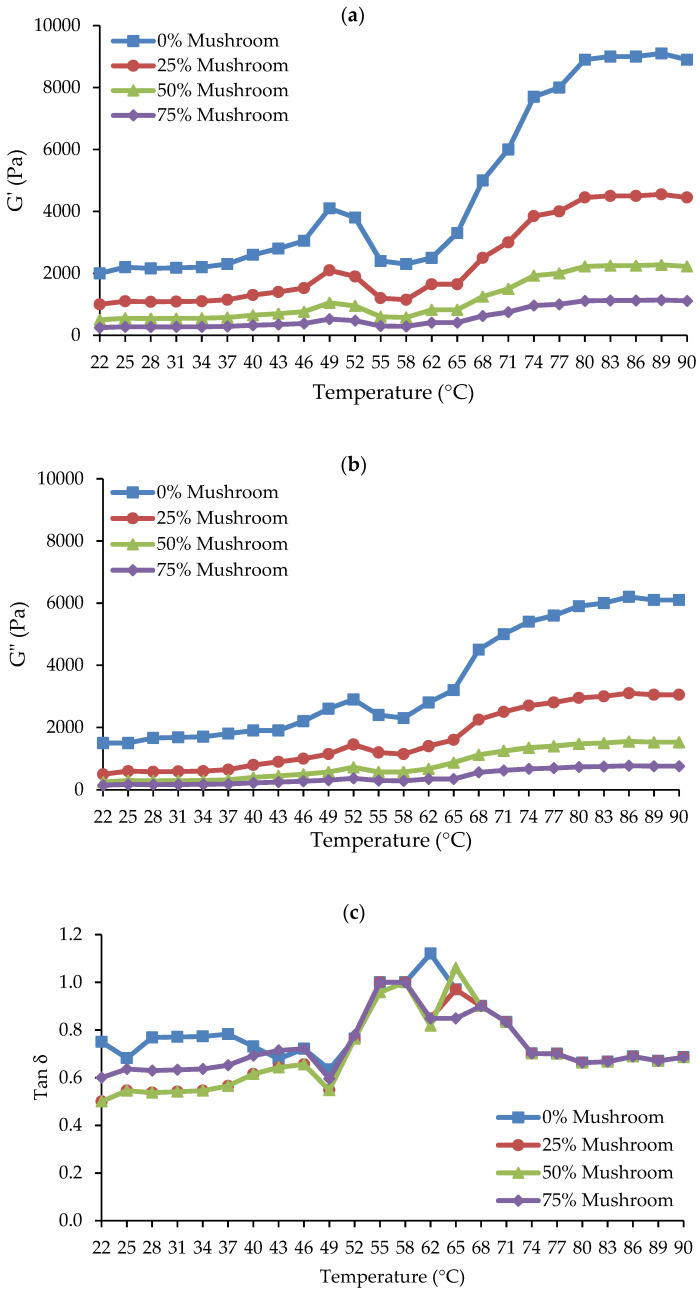
Dynamic rheological properties of chicken meat-mushroom hybrid sols with varying levels of spilt gill mushroom (*Schizophyllum commune*) powder (SGM) substitution (0%, 25%, 50%, and 75%), monitored during heating from 22 °C to 90 °C: (**a**) Storage modulus (G′) showing the elastic behavior of the gel; (**b**) Loss modulus (G″) representing viscous properties; and (**c**) Loss tangent (tan δ = G″/G′) indicating the viscoelastic balance of the gel.

In meat gels, viscoelastic attributes include elasticity and viscosity. Elasticity and viscosity are important rheological parameters that help to understand the structure and behavior of the gel matrix [37]. The elastic modulus (G′), also known as the storage modulus, is a measure of the gel’s solid-like or elastic properties, which represent its ability to store energy when deformed. This increases the gel’s hardness or elasticity. The viscous modulus (G″), also known as the loss modulus, assesses the gel’s liquid-like or viscous properties, indicating its ability to dissipate energy during deformation and is related to flow characteristics or slipperiness [38]. Mushrooms are high in proteins and polysaccharides, which can affect both G′ and G″ when added to meat products in various forms (fresh, powder, or processed), depending on their concentration [14]. Figure 3a–c shows how varying concentrations of SGM powder (0, 25, 50, and 75%) affect the dynamic rheological parameters (G′, G″, and tan δ) of Ligor chicken gel, also known as chicken-mushroom hybrid gels, when heated from 22 °C to 90 °C.

Higher G′ values in meat gels often imply a stronger, more cohesive network that can sustain structure under stress, which is required for a robust, elastic structure [39]. Figure 3a demonstrates that the G′ of chicken-mushroom hybrid sol gradually increased from 22 °C to 40–45 °C. G′ increased steadily until it hit the initial peak at roughly 52 °C, signifying the formation of the initial, weak elastic gelation network. After that, the G′ dropped precipitously from 52 °C to a minimum at roughly 55–58 °C. This is linked to myosin uncoiling from actin (hydrogen bond rupture) and possibly actin filament fragmentation, which raises protein mobility or fluidity because of weakened (deformed) protein-protein interaction [40]. Further heating caused the G′ to rise substantially from about 58 °C to 90 °C, indicating the production of cross-links and protein aggregation that resulted in gel formation, or a stable gel network. Prior research has demonstrated these usual rheological patterns and outcomes in chicken breast meat [41]. The addition of SGM powder to chicken meat reduced the G′ values from the first to the last heat phase when compared to those of the control, suggesting that the use of SGM may reduce the ability of the chicken proteins in the hybrid gels to form gels in a concentration-dependent manner. Consequently, it was shown that the rise in SGM powder concentrations—0, 25, 50, and 75%—was correlated with the decrease in G′. There have been reports of a trend toward a decrease in G′ after plant-based components were added to meat. According to the dynamic rheology of chicken butter, Lin and Barbut [42] found that adding plant proteins (pea, brown rice, faba bean) at 3–12% decreased the G′ during the heating stage (20–72 °C) when compared to the control sample. It was further explained in that study that plant proteins hindered the gelation of meat proteins and postponed the increase in the G′ values of meat butter. Santos et al. [43] showed similar outcomes when they partially substituted plant protein concentrates for beef meat, resulting in weaker gels (decreased hardness and gel strength). Therefore, the G′ value pattern at a 25% SGM powder concentration in the chicken-mushroom hybrid gel may indicate a superior gel to the other treatments with higher SGM powder concentrations. The decrease in G′ values at higher SGM concentrations suggests a reduction in the gel’s elasticity and structural strength, leading to a softer and weaker gel network. This change in rheological properties could influence the product’s suitability for specific culinary applications. For instance, hybrid gels with lower G′ values may be ideal for products requiring a softer texture, such as spreads, pâtés, or plant-based meat alternatives designed to mimic tender textures [44]. However, for applications demanding a firm, elastic structure—such as sausage analogs, structured meat replacements, or restructured meat products—higher SGM concentrations may negatively affect consumer acceptance by reducing gel strength and cohesiveness. Therefore, optimizing SGM concentration is essential to balance textural properties for targeted applications, ensuring both structural integrity and consumer acceptance.

Additionally, greater G″ values in meat gels indicated a softer or less cohesive gel, which is ideal for smooth textures and more prone to deformation [39]. Figure 3b illustrates how the trends of G″ and G′ were comparable. G″ increased quickly and peaked when the temperature rose from 25 °C to 48 °C. After that, G dropped quickly to a minimum at 57 °C. Ultimately, when heated to 90 °C, the G″ rose. The G″ of the chicken-mushroom hybrid gels also considerably decreased as the concentration of SGM powder increased. Amiri et al. [45] reported a correlation between improved water binding of the myofibrillar proteins and increased viscosity. Depending on the concentration, the SGM powder may reduce the protein networks’ ability to hold water. Overall, over the whole heating period of the gel formation, the G″ values of the gel were lower than the G′ values, suggesting that elasticity predominated over viscosity in both the chicken meat system and the chicken meat-mushroom system.

Loss tangent (Tan δ), the ratio of G″ to G′, is a characteristic value for evaluating a substance’s viscoelastic behavior. It impacts the overall strength and stability of the gel [46]. In elastic behavior, tan δ is less than 1, while viscous behavior has tan δ > 1 [32]. In this work, tan δ values were less than 1 at most heating temperatures, and declined with increasing SGM powder content, notably at temperatures below 50 °C (Figure 3c). Tan δ levels in all samples varied significantly between 22–40 °C and 58–68 °C. However, around 68–90 °C, all the gels had the same tan δ values which were lower than 1, suggesting the likelihood of gel formation in all treatments when the thermal gelation was achieved at 90 °C.

### 3.2. Gelling Properties

#### 3.2.1. TPA

TPA-measured textural features are critical for assessing the functional aspects of proteins in gels [47]. These characteristics are essential for selecting high-quality meat products, which influence consumer acceptance [48]. In this investigation, the addition of SGM powder at concentrations of 0, 25, 50, and 75% significantly affected all TPA properties of chicken gel, depending on the concentration used (Table 1). For instance, the hardness decreased in direct proportion to the amount of SGM powder (*p* < 0.05). Compared to the control gel (6.75 N), hybrid gels displayed noticeably reduced hardness values (1.41–3.24 N) (*p* < 0.05). The diluting of muscle proteins by non-meat ingredients with a weaker gel-forming ability may have contributed to the hybrid gel’s decreased hardness, especially at higher concentrations. Gumminess and chewiness followed the same trend as hardness, with SGM-containing gels being less sticky and chewy than the control (*p* < 0.05). Cohesiveness remained stable at 25% SGM but significantly decreased at 50–75% (*p* < 0.05). While 25% SGM had no effect on springiness (*p* > 0.05), higher concentrations (50–75%) significantly reduced it (*p* < 0.05). The 25% SGM hybrid gel maintained a soft yet elastic texture, making it a viable option for texturized products containing active ingredients.

The variations in cohesiveness and chewiness highlighted significant textural changes. At 25% SGM, cohesiveness remained stable, but at 50% and 75%, it declined (*p* < 0.05), weakening the gel’s structural integrity and resilience. Chewiness also decreased with higher SGM levels, contributing to a softer texture. While a softer gel may be desirable for pâtés or spreadable products [44], it may reduce consumer acceptance for structured meat analogs or processed meats requiring firmer textures. Reduced cohesiveness and chewiness affect bite resistance and mouthfeel [49], making SGM concentration a critical factor in achieving the desired texture for specific applications. The textural qualities of the control sample were similar to those of commercial chicken products, however, adding SGM powder resulted in a softer texture, particularly at higher levels. These findings were consistent with previous research, which found that adding edible mushrooms such as grey oyster mushroom (*Pleurotus sajor-caju*), white button mushroom (*Agaricus bisporus*), and straw mushroom (*Volvariella volvacea*) to meat products (e.g., chicken patties, beef paste, and pork sausages) significantly reduced hardness, cohesiveness, gumminess, and chewiness [13,31,50,51]. The bioactive compounds in mushrooms, such as phenolic compounds, were found to interact with meat proteins to form thiol-quinone adducts, which altered the gel’s structure and water-holding capacity (WHC) and produced a softer texture [52,53]. Additionally, residual proteolytic enzymes in mushrooms may break down meat proteins [54,55], whereas dietary fibers in mushrooms may trap fluids, resulting in decreased hardness [56]. Protein-fiber-protein or protein-phenolic-protein interactions could be an aspect of maintaining the gel’s springiness even when its hardness was diminished. In contrast, black jelly mushroom (*Auricularia polytricha*) did not significantly modify the textural qualities of chicken patties, most likely due to its distinct springy-soft texture and the concentration utilized [48]. Similar findings were observed in trials utilizing oyster mushrooms and bamboo shoot fibers in pork and other meat products (beef, buffalo meats, etc.), where the inclusion of mushrooms improved textural qualities [57,58,59,60]. The influence of mushrooms on the textural properties of meat products varied depending on the type of mushroom used, which might be attributed to both the chemical composition and the microstructural/mechanical properties of the mushroom.

Overall, the lower the SGM powder employed, the better the ability to sustain the gel TPA relative to control without SGM. Some TPA measures, such as cohesiveness and springiness, remained unchanged after applying 25% SGM powder, whereas other TPA parameters, such as hardness and gumminess, decreased by almost 2 times and chewiness by nearly 3 times. These alterations were amplified with larger levels of SGM powder. It may be inferred that using 25% SGM powder will result in a soft chicken-mushroom hybrid gel that retains its cohesion and springiness. Furthermore, due to the presence of natural antioxidants in SGM powder, as previously demonstrated [19,24,25], the hybrid gel may be resistant to lipid and protein oxidation during storage to some extent.

#### 3.2.2. Microstructure

**Figure 4:**
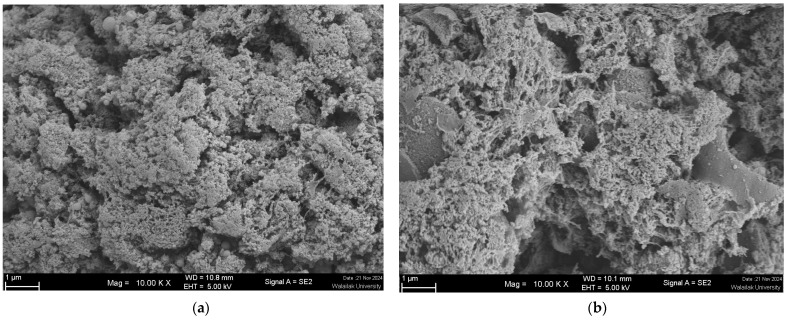

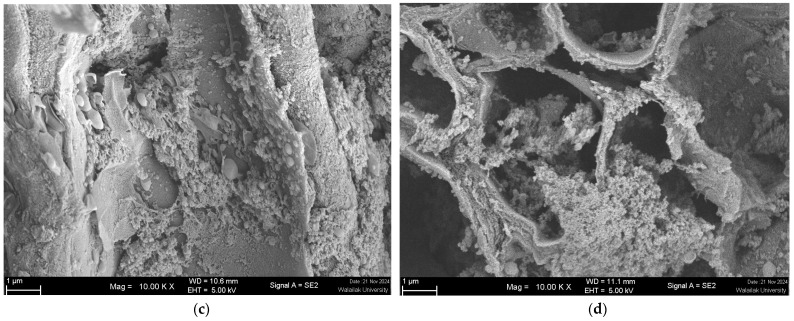
Scanning electron microscopic (SEM) images of chicken meat-mushroom hybrid gels substituted with spilt gill mushroom (*Schizophyllum commune*) (SGM) powder at the level of 0% (Control; (**a**), 25% (**b**), 50% (**c**), and 75% (**d**)) based on the total weight of the meat sample. Magnification: 10,000×, EHT: 5 kV. Highly porous structure with larger voids, indicating a disrupted gel matrix and reduced gel strength.

To better understand the textural changes in gels caused by replacing chicken meat with SGM powder, the gels’ microstructure was documented using SEM (Figure 4a–d). The protein-protein interactions are primarily responsible for heat-induced gelation. Exogenous additives may improve or impede the gelling characteristics of myofibrillar proteins, depending on their ability to produce composite gel and modify intermolecular interactions [61]. The gels incorporated with SGM powder at various concentrations (0, 25, 50, and 75%) showed considerably diverse microstructures, with a propensity toward macropore architectures when SGM powder was applied. Furthermore, the creation of holes on gel structures increased proportionally with increasing SGM powder concentrations. It was observed that the control gel had a dense and compact matrix, which is typical of gels with limited macropore structure. The addition of 25% SGM powder resulted in a hybrid gel with higher porosity and small, evenly dispersed macropores. With 50% SGM powder, the hybrid gel had substantially larger and more numerous macropores, but the hybrid gel with 75% SGM powder exhibited excessive porosity, irregular and larger macropores, and void space.

The formation of larger and more irregular macropores at higher SGM concentrations (50–75%) likely compromises water retention and gel stability (Table 1). Excessive porosity can increase expressible fluid, leading to higher water loss and reduced gel integrity. This weaker gel network may result in phase separation, negatively affecting the product’s texture and sensory properties. To mitigate these effects, optimizing SGM concentration is crucial. Maintaining SGM levels at or below 25% can help balance porosity while preserving gel strength and water-holding capacity. Incorporating hydrocolloids such as carrageenan, xanthan gum, or alginate can enhance water retention and reinforce the gel matrix [62]. Additionally, protein cross-linking using transglutaminase or other agents may improve protein interactions, reducing macropore formation and enhancing gel stability [63]. These strategies can help improve water retention, prevent structural weakening, and maintain the functional and sensory properties of SGM-containing hybrid gels.

Previous research has shown that the holes in the gel matrix can act as water conduits, causing water loss from the gel matrix [64]. According to the results of expressible fluid (see Section 3.2.4) and textural qualities (Table 1) of chicken gels in this investigation, increasing quantities of SGM powder in the formulation resulted in reduced TPA hardness and enhanced expressible fluid (Table 1). Similarly, it has been demonstrated that the production of larger macro holes, less compact and heterogeneous (non-uniform) gel matrix than the control gel, especially when SGM powder at 50 and 75% was utilized. The results were consistent with those reported by Fu et al. [13] for chicken sausage made with white button mushroom and Wang et al. [51] for Cantonese sausage formulated with straw mushroom. In contrast, some studies revealed that incorporating white button mushroom, black jelly mushroom, and bamboo shoot dietary fibers increased the density of structure in meat gel matrix like chicken myofibrillar protein gel and pork butter, resulting in a more uniform, compact, homogeneous, and ordered gel microstructure than the control [48,60,65]. Mushrooms contain dietary fibers that, when combined with meat myofibrillar protein, result in low calorie, low fat, and high fiber meat products due to their ability to form gel networks that hold water and modulate texture [66]. However, their inclusion in meat products should be optimized. In general, our investigation found that adding 25% SGM powder resulted in a chicken-mushroom hybrid gel with compromised TPA and microstructure.

#### 3.2.3. pH

The pH of meat and meat products is a crucial component in determining their quality, as it affects shelf life, texture, color, WHC, and microbial growth [16,67]. Table 1 shows that adding more SGM powder to the gels increased the pH values (*p* < 0.05). When the SGM powder was applied at 0, 25, 50, and 75% concentrations, the pH values were 6.02, 6.34, 6.37, and 6.42. The results were consistent with those of Fu et al. [68], who discovered a significant variation in pH following the addition of various quantities of edible mushroom powder to marinated pork. According to Lee et al. [54], edible mushrooms have pH values ranging from 6.0 to 6.7, depending on their growing conditions and soil type. In contrast, Boylu et al. [16] discovered that adding different levels of oyster mushroom to pork sausage had no significant influence on pH. The pH changes in gel after the addition of SGM powder may be related to the pH of SGM powder and the buffering capacity of chicken meat.

Myofibrillar proteins have an isoelectric point (pI) of approximately pH 5, and increasing or decreasing the pH results in a higher negative or positive charge [69]. Protein gelation is controlled by the pH of the environment; pH levels near the pI but not at the pI frequently create course gel, such as egg white protein, due to encouraging intermolecular interactions between protein molecules [70]. Sun and Holley [71] demonstrated that heat-induced myosin gelation is optimal at pH 6. According to those principles, the control with a pH of 6.02 can produce the gel with the highest hardness (Table 1). However, when SGM powders are added, the gel-forming ability of the chicken-mushroom sol was lower than the control due to the higher pH caused by increasing the SGM powder quantities, as indicated above. The variations in protein subunit compositions and secondary structures, intermolecular interactions, and non-network protein content led to the disparities in gel performance between pH and protein species [72].

#### 3.2.4. Expressible Fluid

Expressible fluid, a crucial gelled food quality measure, is frequently utilized as a WHC indication [47]. A lower expressible fluid suggests that a certain gelled food has a high WHC or a high capacity for lipid retention [73]. Table 1 shows the effects of incorporating SGM powder at 0, 25, 50, and 75% on the expressible fluid in chicken meat gel. According to the findings, the SGM powder-treated sample had a substantially higher expressible fluid than the control gel (*p* < 0.05). Additionally, the expressible fluid in the gels increased as the SGM powder concentration increased (*p* < 0.05). The expressible fluid for the control gel was 10.23%. When the SGM powder was applied at 25%, 50%, and 75%, the expressible fluid increased by 1.8, 2.5, and 3.2 times, respectively. Wan Rosli et al. [50] found that introducing oyster mushrooms to chicken patties at 0, 25, and 50% increased the amount of cooking loss, which is related to higher expressible fluid.

A large percentage of mushrooms was found to have prevented the fibers from forming a three-dimensional matrix with the patties. Similar factors may likely be at play in our investigation. No matter how much more expressible fluid was lost due to the SGM powder, a 25% integration showed the least amount of change in expressible fluid along with appealing textural qualities (Table 1), which might have helped the product be accepted by customers. Our findings contradict with those of Patinho et al. [33] and Fu et al. [13], who found that adding white button mushrooms to beef burgers and chicken sausage, respectively, increased their ability to hold water. According to a description, mushrooms contain substances like polysaccharides that are hydrophilic and have the ability to retain water. However, the type of mushroom, the amount of meat substitute, and the cooking technique can all affect the hybrid meat products’ capacity to gel and, consequently, their WHC.

#### 3.2.5. Color

One of the primary factors influencing customer choice in food products, in particular meat derivatives, is color [74]. Table 1 illustrates how various concentrations of SGM powder (0, 25, 50, and 75%) affect the color characteristics of chicken-mushroom hybrid gels, including lightness (L*), redness-greenness (a*), and yellowness-blueness (b*). The original gray-green color of SGM powder (Figure 1d) utilized in hybrid gel formulations may be the cause of the notable changes in L*, a*, and b* values shown in our results; similar findings have also been reported by Wan Rosli et al. [50], Qing et al. [31], and Lee et al. [54].

In general, the hybrid gel’s color gets darker as the SGM power level rises. Compared to hybrid gels containing SGM powder, the control chicken gel was substantially lighter, as indicated by a greater L* value (*p* < 0.05). In comparison to the control chicken gel (L* value = 77.26), the L* values of the gel with SGM powder ranged from 40.10 to 54.99. While the L* value declined as the concentration of SGM powder increased in all treatments, the gels treated with 25% substitution showed a greater L* value than the other gels treated with mushrooms (*p* < 0.05). Our findings were in line with those of Boylu et al. [16], Yahya and Ting [55], and Fu et al. [13] on the substitution of white button mushrooms (0, 10, 30, 50, and 70%) for chicken emulsion type sausage and the substitution of fresh gray oyster mushrooms for pork and chicken sausage.

The 25% addition of SGM powder to the formulation had no effect (*p* > 0.05) on the a* value, which indicates the redness of the sausage, and was equivalent to the control treatment (Table 1). The a* values of chicken gels containing 50 and 75% SGM powder were lower, indicating a significant (*p* < 0.05) difference from the control and sample containing 25% SGM powder. Wan Rosli et al. [50] found that adding oyster mushrooms to chicken patties at 0, 25, and 50% resulted in comparable or maintained a* values between the control and 25% treated samples. When edible mushrooms including *Sarcodon aspratus*, white button mushroom, and *Lentinula edodes* were added to beef and buffalo patties, the redness index remained comparable to the control [54,59]. The concentration and redox state of the pigment, the physical characteristics of the meat, and the inclusion of non-meat ingredients are some of the factors that affect the color of meat and meat products [75]. The decrease in a* values in hybrid gels containing 50 and 75% SGM powder was also consistent with Boylu et al. [16] and Qing et al. [31], who found that adding oyster mushrooms to beef paste and pork sausage, respectively, decreased the a* value. This was explained by the fact that the final products had less myoglobin due to the meat substitution. Even while chicken meat had less myoglobin than red meat, a similar explanation most likely might have applied to our study’s findings. Therefore, the pigment of the SGM powder added may be the primary cause of the loss of redness in chicken-mushroom hybrid gels. Furthermore, Qing et al. [31] found that polyphenol oxidase, which is present in fresh edible mushrooms, may also have contributed to the lower a* value in beef paste by promoting the oxidation of phenolic compounds and thus influencing the coloring of beef paste. In summary, the current investigation found that a 25% addition of SGM powder produced a promising red color that may affect consumer perception. However, other color parameters, particularly L*, a*, and b* values, were responsible for the final hybrid gel’s overall color.

Incorporating SGM powder into chicken gel formulations reduced yellowness-b* value compared to control gel (Table 1). In fact, the control gel exhibited the highest b* value (13.78) across all mushroom-based gels, with values ranging from 12.79 to 10.71. Among the hybrid gels, the gel with the lowest SGM powder at 25% had a greater b* value. The findings were consistent with previous research, which found that adding oyster mushrooms and other edible mushrooms such as *Sarcodon aspratus*, white button mushroom, and *Lentinula edodes* to chicken, buffalo, and beef patties reduced the b* value of the products [50,54,59]. The higher b* value in the control sample compared to the mushroom treated gels was attributed to a larger amount of chicken sample in the gel [55], which could also apply to our results. In contrast, Boylu et al. [16] and Qing et al. [31] discovered a rise in b* value in pork sausage and beef paste produced with oyster mushroom and other kind mushroom. The increase was ascribed to the color of those mushrooms, which are high in dietary fiber, which increases the b* value of the products. Overall, the amount of SGM powder employed had a significant impact on the discoloration of chicken-mushroom hybrid gel, with the lower SGM powder (i.e., 25%) resulting in a lower darkening degree of the resulting hybrid gel.

## 4. Conclusions

The incorporation of SGM powder into the chicken meat formulation altered key quality characteristics such as texture, color, and water-holding capacity while contributing to sustainability by reducing reliance on animal protein. Optimal formulations, such as the 25% substitution of SGM powder, retained acceptable rheological and gelling properties, making this approach promising for enhancing the functional, nutritional, and environmental attributes of hybrid meat products. These findings suggest that a chicken-mushroom hybrid meat product with SGM powder could be a viable and innovative solution to the health and environmental challenges associated with traditional meat consumption.

However, this study has certain limitations. The absence of sensory evaluation means that the impact of SGM powder on consumer acceptability, including flavor, aroma, and overall eating experience, remains unknown. Additionally, since only basic gel models were tested, further optimization incorporating seasoning ingredients and other functional additives is necessary to enhance palatability. Future research should focus on conducting comprehensive sensory analysis, flavor profiling, and consumer acceptance studies. Moreover, assessing the shelf-life stability of hybrid gels under different storage conditions would be essential for commercial viability. Investigating alternative processing techniques to improve texture and water retention in high-SGM formulations could also be explored to expand potential applications.

## Figures and Tables

**Table 1:** Gelation functionality of chicken meat-mushroom hybrid gel, substituted with spilt gill mushroom (*Schizophyllum commune*) (SGM) powder at the level of 0, 25, 50, and 75% based on the total weight of the meat sample in terms of appearance, texture profile analysis (TPA), pH, expressible fluid, and color.

Parameters	Split Gill Mushroom (*Schizophyllum commune*) Powder Substitution (%)			
	0	25	50	75
Appearance	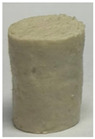	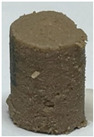	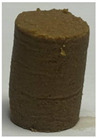	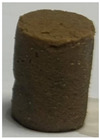
Texture Profile Analysis				
Hardness (N)	6.75 ± 0.27 ^a^	3.24 ± 0.22 ^b^	2.63 ± 0.49 ^b^	1.41 ± 0.01 ^c^
Cohesiveness	0.52 ± 0.05 ^a^	0.44 ± 0.02 ^ab^	0.35 ± 0.04 ^b^	0.35 ± 0.03 ^b^
Springiness (mm)	9.64 ± 0.37 ^a^	8.74 ± 0.51 ^a^	7.59 ± 0.52 ^b^	6.41 ± 0.19 ^c^
Gumminess (N)	3.47 ± 0.23 ^a^	1.95 ± 0.08 ^b^	0.93 ± 0.04 ^c^	0.43 ± 0.07 ^d^
Chewiness (N.cm)	33.54 ± 3.49 ^a^	12.32 ± 0.82 ^b^	7.03 ± 1.62 ^c^	3.14 ± 0.21 ^c^
pH	6.02 ± 0.01 ^c^	6.34 ± 0.03 ^b^	6.37 ± 0.01 ^b^	6.42 ± 0.02 ^a^
Expressible fluid (%)	10.23 ± 1.30 ^d^	18.35 ± 2.14 ^c^	25.65 ± 2.80 ^b^	32.70 ± 9.46 ^a^
Color				
L*	77.26 ± 2.09 ^a^	54.99 ± 2.02 ^b^	48.20 ± 1.31 ^c^	40.10 ± 1.14 ^d^
a*	3.28 ± 0.23 ^a^	3.29 ± 0.10 ^a^	2.88 ± 0.07 ^b^	1.20 ± 0.05 ^c^
b*	13.78 ± 0.15 ^a^	12.79 ± 0.09 ^b^	11.70 ± 0.18 ^c^	10.71 ± 0.53 ^d^

Each reported value represents the mean ± standard deviation (SD) of three replicates. Means in the same row followed by different uppercase letters ^a–d^ are significantly different from each other, as determined by Tukey’s test (*p* < 0.05).

## Data Availability

The original contributions presented in the study are included in the article, further inquiries can be directed to the corresponding author.
